# Nutrition and Diet in Rheumatoid Arthritis

**DOI:** 10.3390/nu14040888

**Published:** 2022-02-19

**Authors:** Maurizio Cutolo, Elena Nikiphorou

**Affiliations:** 1 Experimental Rheumatology Laboratories and Academic Division of Clinical Rheumatology, Department of Internal Medicine DIMI, Postgraduate School of Rheumatology University of Genova, IRCCS San Martino Polyclinic, 16136 Genova, Italy; 2 Centre for Rheumatic Diseases, School of Immunology and Microbial Sciences, King’s College London, London SE5 9RJ, UK; enikiphorou@gmail.com

Rheumatic and musculoskeletal diseases (RMDs) are chronic systemic immune/inflammatory conditions characterized by the interaction between gene predisposition, autoimmunity and environmental factors. A growing scientific interest has focused on the role of nutrition in RMDs, suggesting its significant contribution to the pathogenesis and prognosis of these diseases. The diet can directly modulate the immune response by providing a wide range of nutrients, which interfere with multiple pathways at both the gastro-intestinal and systemic level. Moreover, diet critically shapes the human gut microbiota, which is recognized to have a central role in the modulation of the immune response and in RMD pathogenesis, such as in rheumatoid arthritis (RA). Choosing the ‘right’ diet is therefore crucial and a form of self-management ‘intervention’ that could impact on disease expression, course and outcome [1,2,3].

Several studies have recently suggested that the use of omega-3 and moderate alcohol consumption may have a protective effect on RA development, particularly among smokers or individuals at high risk, as discussed by Alpízar-Rodríguez D et al. in the present Issue [4].

The authors postulate that the microbiota and the intestinal barrier may be a missing link between the various nutritional factors and the development of RA. The modification of microbiota using dietary interventions and focusing on the improvement of the intestinal barrier function may become an important component for “preventive” nutritional strategies. Therefore, as a matter of fact, it seems possible that high salt consumption or sugar sweetened soda, for example, may have a negative effect on gut microbiota, increasing the presence of Prevotella copri strains with higher Branced Chain Amino Acids (BCAA) and Lactobacillus depletion resulting on higher intestinal inflammation [4]. Conversely, omega-3, the Mediterranean diet, probiotics and fiber-rich diets exert positive effects on gut microbiota, favoring Prevotella Copri strains with higher potential for carbohydrate degradation and increased short-chain fatty acids (SCFA) synthesis, leading to less intestinal inflammation in RA patients [4]. It is suggested that longitudinal cohort studies during the preclinical phases of RA studying dietary patterns and microbiota changes concurrently should be considered to better understand the causality of these associations [4].

On the other hand, short-chain fatty acids that are gut-bacteria-derived metabolites exert regulatory functions on adaptive immune responses. This is despite their influence on inflammation driven by innate immunity remaining poorly investigated. Friščić et al. report in this Issue that Dietary Derived Propionate seems to regulate pathogenic fibroblast function and ameliorates experimental arthritis and inflammatory tissue priming [5].

The authors show that propionate treatment in drinking water or upon local application into the joint reduced experimental arthritis and decreased the inflammatory tissue priming mediated by synovial fibroblasts. In addition, the incubation of synovial fibroblasts with propionate or a physiological mixture of short-chain fatty acids interfered with production of inflammatory mediators, cell migration and induced immune-regulatory fibroblast senescence [5].

Additionally, nutrients, including trace elements such as the essential metal zinc (Zn2^+^) and the non-essential metal cadmium (Cd2^+^) have roles in RA as effectors of the immune system, inflammation and metabolism, as reported by Frangos T. et al. [6].

Despite both metal ions being redox-inert in biology, they affect the redox balance, and it has long been known that zinc decreases in the blood of RA patients. Zinc, as a cofactor in over 3000 human proteins and signaling ions, affects many pathways relevant for arthritic disease, including osteoarthritis, and as a signaling ion, zinc also affects many pathways involved in the arthritic process [6].

On the contrary, cadmium interferes with zinc’s activities, and there is increased uptake in the presence of zinc deficiency. Interestingly, cadmium exposure through inhalation is now recognized in the activation of macrophages to a pro-inflammatory state and suggested as a trigger of a specific form of nodular RA. The authors suggest that both metal ions, namely, zinc and cadmium, should be monitored routinely in RA [6].

Related to diet, Edefonti V. et al. report in the present Issue on the relationship between a posteriori dietary patterns (DPs)—representing current dietary behavior—and disease activity in RA patients [7]. In this cross-sectional study of 365 RA patients, prevalent DPs were identified through principal component factor analysis on 33 different nutrients. The authors identified five DPs (~80% variance explained), and among them, vegetable unsaturated fatty acids (VUFA) and animal unsaturated fatty acid (AUFA) DPs were inversely related to DAS28 (Disease Activity Score on 28 joints) in the overall analysis, as well as in the more severe or long-standing RA subgroups [7]. Interestingly, the highest score reductions (VUFA: 0.81, AUFA: 0.71) were reached among the long-standing RA patients. In addition, the SDAI (Simplified Disease Activity Index) was found to be inversely related with these DPs in subgroups only. The study seems to show that scoring high on DPs based on unsaturated fats, even from different sources, may provide independent contribution of clinical relevance on RA disease activity [7].

Evidence generally suggests that an inappropriate diet may be a possible facilitator of RMDs due to both the direct pro-inflammatory properties of some nutrients as well as the indirect action on insulin resistance, obesity and associated co-morbidities. However, most of the research to date has focused on RA, with scarce evidence in other disease areas. Yet, many rheumatic diseases share common pathophysiological mechanisms and inflammatory pathways. Alunno et al. undertook a comprehensive review on the topic of dietary choice and impact in systemic lupus erythematosus (SLE), enriching the literature in this disease area [8]. The authors concluded that studies on experimental SLE and on SLE patients suggest a positive role for some nutrients, such as polyunsaturated fatty acids (PUFAs) and moderate alcohol intake, in line with what is observed in RA. More evidence is also needed in SLE to understand potential differences with other RMDs [8]. Generally, the Mediterranean diet is highly recommended in RMDs due to its anti-oxidant and anti-inflammatory properties and may be suggested as the preferred dietary pattern in both RA and SLE. On a more overarching note, nutritional counselling in SLE, as with other RMDs, should be performed at least to prevent obesity, malnutrition and CV risk.

A further study by Paolino et al. investigated the impact of gastrointestinal (GI) involvement on bone microarchitecture in systemic sclerosis (SSc). GI involvement in SSc is characterised by the atrophy of the smooth muscle and small bowel hypomotility, overall coming from autonomic nerve dysfunction [9]. These alterations significantly affect gut transit and nutrient absorption, culminating in nutritional deficits induced by malabsorption that consequently also led to bone alterations. In summary, this pilot study demonstrated that in malnourished SSc patients (2015 ESPEN criteria: FFMI < 15 kg/m^2^), an altered bone status significantly correlated with GI involvement [9].

Much attention in the recent literature has also focused on alcohol, one of the highest consumed and abused worldwide substances. However, and paradoxically, alcohol has also been shown to have a protective effect against the development of autoimmune diseases such as type 1 diabetes, multiple sclerosis and from the RMDs, SLE and RA. However, the amount of alcohol consumption becomes relevant in this regard. In their review in this Issue, Azizov et al. report that heavy or moderate alcohol consumption can affect intestinal barrier integrity, as well as the gut microbiota, possibly contributing to RA [10].

On the other hand, a systemic increase in acetate U (its metabolite) negatively affects humoral immune response, diminishing TFH cell as well as professional antigen-presenting cell (APC) function. Alcohol consumption seems to exert effects on the efficacy of vaccinations but also induce protection against autoimmune diseases. In any case, the mechanism of alcohol’s negative effects on the immune system is multivariate, as the authors conclude [10].

It is important, however, to remember that the amount of alcohol consumption is relevant, as also supported by a meta-analysis of a total of 195,029 participants, including 1878 RA cases, which showed that low to moderate alcohol consumption is inversely associated with the development of RA in a dose-dependent, time-dependent and sex-dependent manner [11].

As mentioned, beverages have a key role within the mosaic of autoimmunity in RA and potential to alter the gut microbiota, leading to downstream effects on inflammatory systemic pathways. The review in the present Issue by Dey et al. underlines that the molecular contents of beverages, including coffee, tea and wine, have been found to interfere with immune signaling pathways. Some have beneficial effects for disease progression and others not [12]. Positive effects for all fresh juice fruits are reported. While there is increasing work focusing on the role of beverages in RA, the integration of discussions around diet and lifestyle in the management of RA patients remains sparse [12].

In this Issue, we also get to understand the potential role of spice supplementation in RA through the work of Letarouilly et al. [13]. The effects of spices such as curcumin, ginger, saffron and cinnamon have been searched in a systematic literature review (SLR) of randomized controlled trials (RCTs). Across six studies assessing the use of spice supplementation only in RA patients (one on garlic supplementation, two on curcumin, one on ginger, one on saffron and one on cinnamon supplementation), the authors conclude that that garlic, ginger, cinnamon or saffron supplementation was associated with a decrease in RA clinical activity. However, one needs to appreciate that several points limit the external validity of these findings due to high risk of bias associated with these studies [13].

Finally, in this Issue and through a systematic review and meta-analysis of randomized controlled trials, Nguyen et al. evaluated the role of oral Vitamin D (D hormone) supplementation in inflammatory rheumatic diseases [14]. Of 606 articles screened, 13 were included in a qualitative data synthesis: 8 studied vitamin D supplementation, 2 assessed vitamin E supplementation, 2 folic acid and 1 vitamin K, all of them in RA patients. No studies in SpA or PsA were selected. Oral vitamin supplementations were not associated with a significant reduction in RA activity (DAS-28 or pain) or RA flares [14]. However, vitamin D, with inherent immunosuppressive effects being a steroid hormone, should at least be given to RA patients with known vitamin D deficiency to prevent musculo-skeletal complications [15]. The highly selective inclusion research criteria of this systematic review suggest caution prior to drawing key conclusions on what is already a controversial subject on the potential role for vitamin D in RA [15,16].

In conclusion, through this Special Issue and severalrelevant and interesting topics presented, we cannot dismiss the important role of nutrition in RA and other rheumatic diseases. An appropriate and well-balanced diet does not replace the pharmacological management of disease but should at least be used as an adjuvant to medical treatment, with most evidence supporting this coming from studies in RA [17]. Dietary counseling together with disease-modifying anti-rheumatic drugs should both form part of the early management of RA.
